# Compatibility of synthetic and biological pesticides with a biocontrol agent *Phytoseiulus longipes* (Acari: Phytoseiidae)

**DOI:** 10.1007/s10493-024-00926-3

**Published:** 2024-06-13

**Authors:** Patrice Jacob Savi, Gilberto José de Moraes, Fabien Cossi Charles Hountondji, Christian Nansen, Daniel Júnior de Andrade

**Affiliations:** 1 Department of Entomology and Nematology, University of California, Davis, CA USA; 2 Departamento de Fitossanidade, Universidade Estadual Paulista (UNESP), Faculdade de Ciências Agrárias e Veterinárias, Universidade Estadual Paulista - Laboratório de Acarologia (AcaroLab), Jaboticabal, SP 14884-900 Brasil; 3 Departamento de Entomologia e Acarologia, Escola Superior de Agricultura “Luiz de Queiroz”, Universidade de São Paulo, Piracicaba, SP 13418-900 Brasil; 4 Faculté d’Agronomie, Université de Parakou, Parakou, BP 123 Benin

**Keywords:** Phytoseid mites, Augmentative releases, Conservation biological control, IPM *Tetranychus evansi*, Pesticide selection, Residual effect

## Abstract

*Phytoseiulus longipes* is a predatory mite of *Tetranychus evansi*, which is an invasive pest in Africa and elsewhere. The introduction of this predator in Africa has considerable potential, but little is known about the compatibility of *P. longipes* with commonly used pesticides. Here, we examined lethal and sublethal effects of two pyrethroids (cypermethrin and deltamethrin), two organophosphates (dimethoate and chlorpyrifos), one nicotinoid (imidacloprid), two acaricides (propargite and abamectin), two naturally derived pesticides (oxymatrine and azadirachtin), and one entomopathogenic fungal-based formulation (*Hirsutella thompsonii*) on *P. longipes* eggs and adults. The pesticides were sprayed at their maximum recommended concentrations. Topical exposures to azadirachtin, imidacloprid, propargite, abamectin, oxymatrine, and *H. thompsonii* significantly reduced the net reproductive rate (*R*_0_), intrinsic rate of increase (*r*) and finite rate of increase (*λ*)of *P. longipes*. Pesticide lethal and sublethal effects on the predator were summarized in a reduction coefficient (*E*_*x*_) for the classification based on IOBC toxicity categories. Results revealed that Azadirachtin and *H. thompsonii* were slightly harmful effects to adults. Imidacloprid, propargite, abamectin, and oxymatrine were moderately harmful to both eggs and adults. Residual persistence bioassays revealed that 4-day-old residue of azadirachtin had no harmful effect on the predator. Abamectin, oxymatrine, and *H. thompsonii* became harmless to it 10 days post-spraying, and propargite and imidacloprid were considered harmless after 20 days. Cypermethrin, deltamethrin, dimethoate, and chlorpyrifos were highly harmful to both eggs and adults, persistence remaining high even after 31 days of application. These findings provide valuable insights into decision-making when considering *P. longipes* for use in IPM programs.

## Introduction

Predatory mites from the Phytoseiidae family are widely recognized in various agricultural settings as effective biocontrol agents of herbivorous mites, and small insect pests including the western flower thrips [e.g. *Frankliniella occidentalis* (Pergande) (Thysanoptera: Thripidae)] and whiteflies [e.g. *Bemisia tabaci* (Gennadius) (Hemiptera: Aleyrodidae)] (McMurtry 2013; Knapp et al. 2018; Zacarias et al. 2019). Their short life cycle allows for multiple generations within a single growing season, increasing their effectiveness (Abad-Moyano et al. 2009; Knapp et al. 2018). They have also been shown to be effective in mitigating damage when facing the challenge of managing invasive pest mites (Yaninek et al. 1998; Sato et al. 2012). A recent example highlighting the importance of this family involves the use of *Phytoseiulus longipes* Evans. This species found in southern Brazil and northern Argentina, has been particularly promising in recent efforts to combat the red spider mite, *Tetranychus evansi* Baker & Pritchard (Acari: Tetranychidae), an invasive pest in tomato crop and many other solanaceous in some countries in Europe, Asia and Africa (Silva et al. 2010; Ferrero et al. 2011; McMurtry 2013; Savi et al. 2021a, b). The effectiveness of *P. longipes* in controlling *T. evansi* has generated considerable interest in introducing it to Africa, where this invasive pest has caused yield reductions of up to 90% in tomato crops (Navajas et al. 2013; Azandémè-Hounmalon et al. 2015; Savi et al. 2019). *Phytoseiulus longipes* has also been found to be potentially useful for controlling other *Tetranychus* species including *Tetranychus urticae* Koch (Acari: Tetranychidae) (Ferrero et al. 2011; McMurtry 2013). Furthermore, it has shown resilience across a wide range of temperature (Ferrero et al. 2007), making it a suitable candidate for countries and continents affected by *T. evansi* in solanaceous crops.

However, *T. evansi* is often found associated with other pests, some of which have been controlled with the use of pesticides, which in turn may affect that predator, hindering its successful establishment, conservation, or augmentation efforts in IPM. Therefore, it is crucial to assess the potential side effects of pesticides commonly employed in IPM programs on *P. longipes* in areas where the use of the latter is planned. These potential side effects include both direct effects (mortality) and indirect effects (life history traits, such as development time, fecundity, fertility, longevity, sex ratio, predation rate, mobility, orientation and feeding activity) (Desneux et al. 2007; Biondi et al. 2015; Kim et al. 2018; Duso et al. 2020). Furthermore, when evaluating pesticide compatibility, it is important to consider the exposure route related to topical exposure or residual contact (Kim et al. 2018; Bergeron and Schmidt-Jeffris 2020; Duso et al. 2020).

The “Pesticides and Beneficial Organisms” working group of the International Organization for Biological Control (IOBC) has proposed a sequential testing exposition scheme, which is has been used to assess the impact of pesticides on non-target species (Hassan et al. 1994; Van de Veire et al. 2002; Wanumen et al. 2016). This approach categorizes compounds into four toxic classes, ranging from harmless to harmful, and involves sequential testing, starting from laboratory settings and progressing to semi-field or field evaluations, if necessary. Applications of life tables are also essential in evaluation of potential side effects of pesticides (Stark and Banks 2003; Zanardi 2017; Shahbaz et al. 2019; Duso et al. 2020).

Due to the limited understanding of *P. longipes* susceptibility to pesticides, this study aimed to evaluate the lethal and sublethal effects of seven synthetic pesticides and three biopesticides commonly used in Western African tomato crop systems on *P. longipes*, following IOBC approach and considering the effects on life table parameters. The results of this study are expected to enhance our comprehension of the compatibility between *P. longipes* and commonly employed pesticides for tomato pest management. Additionally, the findings will help to determine appropriate timelines for the release of the predator in potential augmentative biological control after pesticide applications.

## Materials and methods

### Mites

*Tetranychus evansi* colony used in this study was established on tomato plants (*Solanum lycopersicum* L. var. TLCV15) in a screen house at São Paulo State University, Jaboticabal Campus, Brazil. *Phytoseiulus longipes* colony was established using specimens collected from *S. lycopersicum*, *Solanum americanum* Mill and *Brugmansia suavolensis* L. (Solanaceae) in the urban area of Uruguaiana, Rio Grande do Sul state, southern Brazil (29°49’48.0” S 57° 06’04.0"W 68 m above sea level and 29°45’12.0"S 57°04’31.0"W 61m above sea level). After confirming the identity of *P. longipes* (de Moraes et al. 2004) using a Nikon Eclipse E200 phase-contrast compound microscope, colonies of the predator were maintained on a synthetic plate (Paviflex®; 22 × 15 cm) resting on a foam mat in a plastic tray (25 × 17 × 9 cm). Predatory mites were fed daily ad libitum with leaflets of TLCV15 tomato genotype infested with all *T. evansi* stages. The leaflets were excised, and their petioles were inserted into a ball of cotton wool in contact with the mat. The mat was maintained wet by daily addition of deionized water, to maintain the turgidity of the leaflet and to prevent mites from escaping. Trays were kept in a climate-controlled chamber at 25 ± 1ºC, 70 ± 10% relative humidity, and a 12-hour photoperiod.

### Chemicals

Seven synthetic pesticides belonging to different chemical families and three biopesticides commonly used in horticultural crops were evaluated for their effects on *P. longipes* eggs and adults. The pesticides were assessed at their maximum field-recommended concentrations (milligrams of active ingredient per liter of water), as registered by the Brazilian Ministry of Agriculture, Livestock, and Food Supply (Agrofit 2021). The evaluated pesticides included two sodium channel modulators: cypermethrin at 62.5 mg.L^− 1^ (Cipermetrina Nortox 25% EC, Nortox Sa Arapongas– PR, Brazil) and deltamethrin at 25.0 mg.L^− 1^ (Decis 2.5% EC, Bayer S.A. SP, Brazil); two acetylcholinesterase inhibitors: chlorpyrifos at 450 mg.L^− 1^ (Sabre 45% EW, Dow AgroScience Industrial, Barueri, SP, Brazil) and dimethoate at 400 mg.L^− 1^ (Dimetoato Nortox 50% EC, Nortox, Arapongas, PR, Brazil); one nicotinic acetylcholine receptor competitive modulator: imidacloprid at 100 mg.L^− 1^ (Provado 20% SC, Bayer S.A. SP, Brazil); one inhibitor of mitochondrial ATP synthase: propargite at 360 mg.L^− 1^ (Omite 72% EC, UPL do Brasil– Indústria e Comércio de Insumos Agropecuários S.A.); one glutamate-gated chloride channel allosteric modulator: abamectin at 3.6 mg.L^− 1^ (Vertimec 1.8% EC, Syngenta Proteção de Cultivos Ltda, SP, Brazil); two naturally derived pesticides: oxymatrine at 2.0 mg.L^− 1^ (Matrine 0.2% SL, Dinagro Agropecuária Ltd., Ribeirão Preto, SP, Brazil), which targets nicotinic acetylcholine receptors and sodium channels (Ali et al. 2017; de Andrade et al. 2019), and azadirachtin at 24 mg.L^− 1^ (Azamax®EC, 1.2% w/v, UPL do Brasil– Indústria e Comércio de Insumos Agropecuários S.A., Ituverava, SP, Brazil), inhibidor of the release of protoraxicotrophic hormone (Mordue and Nisbet 2000); and one entomopathogenic fungal-based formulation: *Hirsutella thompsonii* Fisher at 8.0 mg.L^− 1^ (6.0 × 102 CFU.mL^− 1^) (Skupa-Mite 0.4% SL, Maneogene Agrociências S.A. Jaguariúna– SP, Brazil). Deionized water constituted the control treatment.

### Experimental units

**Fig. 1:**
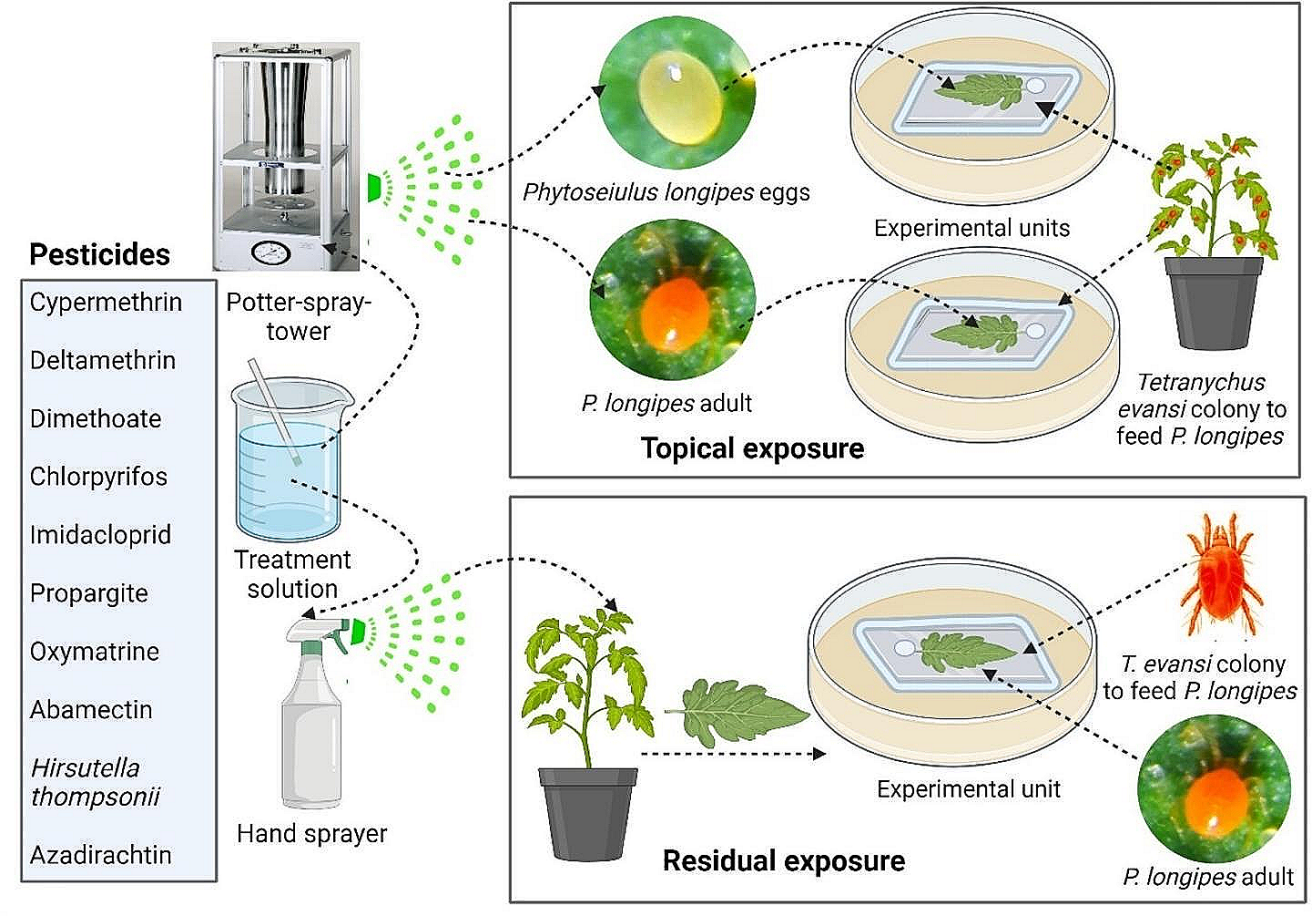
Schematic representation of experimental design

Each experimental unit consisted of a synthetic plate (Paviflex®; 15 mm × 20 mm) placed onto a 1-cm-thick foam mat layer inside a Petri dish (15 × 120 mm) according to the method described by Savi et al. (2021a). The mat was kept wet by adding deionized water daily. A tomato leaflet, with its petiole inserted into a wet cotton wool strip for turgidity, was placed on the synthetic plate to host the tested predator. To prevent mites from escaping, the edge of plate was sealed with a strip of cotton wool, to ensure contact with the mat (Fig. 1).

### Topical exposure of *Phytoseiulus longipes* eggs and adults to the pesticides

Separate experiments were conducted under the same conditions mentioned for the predator stock colony. For the egg experiment, 50 newly laid eggs (< 6 h old) were collected with a brush and transferred next to each other onto a Petri dish. The dish was then subjected to one of the treatments, using a Potter tower (Burkard Scientific Co., Uxbridge, United Kingdom) calibrated at 27.6 kPa. A standard volume of 2 mL of the solution (or deionized water) was sprayed onto the patch containing the eggs, resulting in a deposition of 1.8 ± 0.1 mg fresh residue cm^− 2^. Eggs treated were then individually placed in a separate experimental unit (Fig. 1), following the method described in item 2.3. A mixture of all stages of *T. evansi* that had not been exposed to pesticides was daily provided *ad libitum* to each predator as food. Survival rates, duration of developmental stages (egg, larvae, nymphs - pooling protonymphs and deutonymphs) and fecundity were observed daily under a stereomicroscope following the methodology described by Savi et al. (2021a). Furthermore, male, and female longevities were estimated. Predatory mites that did not react when touched with a fine brush were considered dead. To assess the fertility of eggs laid by females that had reached the adult stage, 10 eggs (< 6 h old) were selected daily per treatment from the first five days of fecundity assessments. These eggs were placed in new experimental units, and daily counts were performed to determine the number of emerged larvae. Eggs that did not hatch within 4 days were considered dead. a reduction coefficient (Ex) ${E}_{X}=100-\left(100-{M}_{c}\right)*{R}_{1}*{R}_{2}*{R}_{3}$ was calculated for each treatment based on mortality and sublethal effects (fecundity, fertility of females, and longevity), according to the adapted formula of Biondi et al. (2012). Mc represents corrected mortality, which was calculated using the Henderson and Tilton (1955) formula.

$$ {\text{M}}_{\text{C}}=\frac{100\text{*} (1- \text{n} \text{i}\text{n} \text{C}\text{o} \text{b}\text{e}\text{f}\text{o}\text{r}\text{e} \text{t}\text{r}\text{e}\text{a}\text{t}\text{m}\text{e}\text{n}\text{t} \text{*} \text{n} \text{i}\text{n} \text{T} \text{a}\text{f}\text{t}\text{e}\text{r} \text{t}\text{r}\text{e}\text{a}\text{t}\text{m}\text{e}\text{n}\text{t})}{\text{n} \text{i}\text{n} \text{C}\text{o} \text{a}\text{f}\text{t}\text{e}\text{r} \text{t}\text{r}\text{e}\text{a}\text{t}\text{m}\text{e}\text{n}\text{t} \text{*} \text{n} \text{i}\text{n} \text{T} \text{b}\text{e}\text{f}\text{o}\text{r}\text{e} \text{t}\text{r}\text{e}\text{a}\text{t}\text{m}\text{e}\text{n}\text{t})}$$

R1 represents the ratio of eggs laid by emerged females from eggs treated with T and Co. R2 represents the ratio of hatched larvae from emerged females from eggs treated with T and Co. R3 represents the ratio of longevity between eggs treated with T and Co. n is the number of live individuals, Co is the control group, and T is the treatment group. E_*x*_ values were compared to the IOBC/WPRS laboratory ecotoxicological test standards, proposed by Sterk et al. (1999) and categorized as follows: I, harmless (E < 30%); II, slightly harmful (30 < E < 79%); III, moderately harmful (80 < E < 99%); IV, harmful (E > 99%).

In the adult experiment, 50 newly emerged mites were used per treatment (25 females and 25 males, < 24 h old), dividing them into five groups, each with five females and five males. Each group was confined to a tomato leaflet with the abaxial side facing up placed on a layer of water-saturated cotton wool in a Petri dish (12 cm in diameter). To prevent the predators from escaping, the leaflet edges were covered with a strip of cotton wool, and some *T. evansi* eggs were added. Subsequently, the leaflets with *P. longipes* adults were sprayed as previously described for the experiments with eggs, using a Potter tower, and maintained in the same unit for 24 h, to evaluate mortality. Surviving adults were separated by gender, with a couple transferred to each experimental unit. Daily observations under a stereomicroscope were conducted to evaluate pesticide effects on fecundity, longevity, and female fertility, following the same procedure described for the egg experiments. Based on the corrected adult mortality rates within 24 h after treatments and sublethal effects (fecundity, fertility, longevity), a reduction coefficient E_x_ was calculated using the same procedure described for the *P. longipes* egg stage. It was then compared to the IOBC/WPRS laboratory ecotoxicological test standards.

### Residual effect and duration of the pesticide’s harmful activity to *Phytoseiulus longipes*

The pesticide residual effects on *P. longipes* adults were assessed using 30-day-old tomato plants of variety TLCV15, grown according to the method described by Savi et al. (2021b). The pesticides were applied on plants, using a hand sprayer (Brudden® Practical; Brudden Equipamento Ltda, Pompeia, São Paulo, Brazil) until the runoff stage (Fig. 1). Each treatment consisted of five replicates, each replicate corresponding to one plant. The plants were arranged in a fully randomized design within a screen-house, under the following conditions: temperature 26.7 ± 0.3ºC, relative humidity 59 ± 1%, and natural light conditions (approximately 10 h of daily light). One leaflet was removed from the median third of the canopy of each plant at 4, 10, 20, and 31 days after spraying. Each leaflet was used to prepare an experimental unit, as described in item 2.3. Ten six-day-old adult *P. longipes* (five females and five males) were introduced into each unit. These predators were provided with surplus untreated *T. evansi* (in all stages) as prey. After 72 h of exposure, the mortality, oviposition, and viability of the predators were recorded. The trials continued until the pesticides were deemed harmless or for up to one month after treatment, based on the criteria established by the International Organization for Biological Control, Western Palearctic Region Section (IOBC/WPRS) (Hassan et al. 1994). For each treatment, a reduction coefficient (Ex) was calculated to estimate the harmfulness of the pesticide. The toxicity categories defined by the IOBC/WPRS were used to classify the persistence of the pesticides: A (short-lived: <5 days), B (slightly persistent: 5–15 days), C (moderately persistent: 16–30 days), and D (highly persistent: >30 days).

### Data analysis

The development time, survival rate, reproduction, and life table parameters following exposure of the eggs to the treatments were estimated using an age-stage two-sex life table model developed by Chi (2022) available at http://140.120.197.173/ecology/Download/Twosex-MSChart.rar. For treatments with immature mortality below 85%, the net reproduction rate (*R*_0_), intrinsic rate of increase (*r*), finite rate of increase (*λ*), and average generation time (*T*) were estimated. Standard errors (SE) were calculated using the bootstrap procedure with 100,000 random resamplings. Paired bootstrap tests (B = 100,000) with a 95% confidence interval were conducted to compare differences between treatments (Efron and Tibshirani 1994; Wei et al. 2020).

Data on adult *P. longipes* exposed directly to the treatments and the residual persistence data were analyzed using a Generalized Linear Model (GLM) with quasi-binomial, quasi-Poisson, and Gaussian distributions for proportion (adult mortalities and female fertility), counts (fecundity), and duration (female and male longevities) (Nelder and Wedderburn 1972). The fit quality was assessed through a half-normal graph with a simulation envelope (Hinde and Demétrio 1998). If significant differences were found between treatments, post-hoc Tukey’s tests (*p* < 0.05) were performed using the “glht” function of the “multcomp” package with adjusted p-values. All analyses were conducted using R v3.6.1.

## Results

### Topical exposure toxicity

#### Life table parameters of *P. longipes* after topical treatment of eggs

In treatments with chlorpyrifos, cypermethrin, and dimethoate, egg hatchability decreased to 0, 6, and 48%, respectively (Table 1). Deltamethrin, propargite, *H. thompsonii* and oxymatrine slightly reduced egg-hatching rates to ~ 72–84%. No significant difference was observed between abamectin, imidacloprid, and control (~ 88–98% versus 100%). Abamectin prolonged the egg stage, whereas cypermethrin, deltamethrin, dimethoate, oxymatrine, propargite, and *H. thompsonii* shortened it; azadirachtin and imidacloprid had no effect on egg stage compared to the control. All treatments, except those with abamectin and azadirachtin, caused significant larval mortality. All treatments caused significant increases in larval development time. Most of the treatments dropped nymphal survival rates below 40%, except those with Abamectin, oxymatrine, azadirachtin, and imidacloprid, which lowered it to 50–60% compared to the control (94%). All treatments caused significant increases in nymphal development time except those with azadirachtin, oxymatrine, *H. Hirsutella*. Except for azadirachtin, oxymatrine, and *H. thompsonii*, all other treatments significantly increased immature development time.

**Table 1:** Mean duration (days ± SE) and survival (%) of immature stages of Phytoseiulus longipes when eggs were treated with pesticides; initial number of eggs for each treatment (replicates) = 50

Treatment	Dose used (mg i.a. L^− 1^)	Eggs			Larvae			Nymphs		Pre-adult duration (days)
		Hatching (%)	Duration (days)		Survival (%)	Duration (days)		Survival (%)	Duration (days)	
Control	-	100.0 ± 0.0a (50)	1.64 ± 0.04b		100.0 ± 0.0a (49)	0.58 ± 0.03c		94.0 ± 3.4a (47)	2.27 ± 0.1 cd	4.5 ± 0.1d
Abamectin	3.6	98.0 ± 0.0 a (49)	2.03 ± 0.07a		90.0 ±.2ab (45)	0.83 ± 0.04b		60.0 ± 6.9b (30)	2.43 ± 0.3bc	5.2 ± 0.2ab
Azadirachtin	24	87.2 ± 4.8ab (41)	1.62 ± 0.07b		92.7 ± 4.1ab (38)	0.74 ± 0.1b		48.9 ± 7.3bc (23)	2.02 ± 0.1e	4.6 ± 0.1 cd
Chlorpyrifos	450	-	-		-	-		-	-	-
Cypermethrin	62.5	6.1 ± 3.4d (4)	1.42 ± 0.08c		-	-		-	-	-
Deltamethrin	25	72.0 ± 6.3b (36)	1.39 ± 0.08c		47.2 ± 8.2d (17)	1.0 ± 0.0a		8.0 ± 3.6d (7)	3.14 ± 0.3a	5.3 ± 0.1a
Dimethoate	400	48.0 ± 7.0c (24)	1.42 ± 0.1 c		83.3 ± 7.6bc (20)	1.0 ± 0.0a		14.0 ± 4.8d (4)	2.5 ± 0.3b	4.8 ± 0.2bc
Imidacloprid	100	95.6 ± 2.9a (44)	1.75 ± 0.08b		88.6 ± 4.8b (39)	0.76 ± 0.04b		45.7 ± 7.3bc (21)	2.8 ± 0.2ab	5.5 ± 0.2a
Oxymatrine	2	76.4 ± 5.4b (38)	1.20 ± 0.07d		86.8 ± 5.5b (26)	0.74 ± 0.04b (33)		78.7 ± 7.2bc (26)	2.37 ± 0.1c	4.5 ± 0.1d
Propargite	360	78.0 ± 5.8b (39)	1.23 ± 0.07d		51.3 ± 7.9d (20)	1.0 ± 0.0a		16.0 ± 5.1d (8)	2.9 ± 0.12a	5.1 ± 0.1b
*Hirsutella thompsonii*	8	79.6 ± 5.7b (40)	1.32 ± 0.08 cd		74.4 ± 7.0c (29)	0.9 ± 0.1a		38.8 ± 6.9c (19)	2.16 ± 0.1d	4.6 ± 0.2 cd

In each column, means followed by the same letter do not differ from each other. Standard errors (SE) were estimated using 100,000 bootstraps and means were compared using the paired bootstrap test at 5% significance. The values in the brackets represent the number of individuals surviving at each stage

Total fecundity was significantly higher in control than other treatments (Table 2). No significant difference was observed in fertility between control and treatments except for that of propargite (GLM with binomial distribution: F = 3.62; df = 6, 49; *p* = 0.002). All treatments, except abamectin, significantly reduced female longevity. All treatments significantly reduced male longevity except with those of abamectin, imidacloprid, oxymatrine, and *H. thompsonii*. According to the reduction coefficient (Ex values) and IOBC/WPRS classification, chlorpyrifos, dimethoate, cypermethrin, and deltamethrin were classified as highly harmful (class IV). Abamectin, azadirachtin, imidacloprid, oxymatrine, propargite, and *H. thompsonii* were deemed moderately harmful (class III) to *P. longipes* eggs. Abamectin, azadirachtin, imidacloprid, oxymatrine, propargite, and *H. thompsonii* treatments caused significant reduction in the net reproduction rate (*R*_0_), intrinsic rate of increase (*r*) and finite rate of increase (*λ*) compared to control (Table 3). Eggs exposed to propargite had a higher average generation time (*T*) than both the control and other treatments. Exposure of *P. longipes* to chlorpyrifos, dimethoate, cypermethrin, and deltamethrin treatments caused nearly 100% mortality of immature, which hindered the estimation of life table parameters.

**Table 2:** Corrected mortality of immature stage and sublethal (total fecundity, fertility, and female and male longevities) effects, reduction coefficient and IOBC/WPRS toxicity categories of pesticides applied on eggs of Phytoseiulus longipes. Initial number of eggs = 50

Treatment	Dose used (mg i.a. L^− 1^)	Corrected mortality of immature stage (M_c_%)	Effect on reproduction (Er)			Longevity		Ex ^b^	IOBC class ^c^
			Total fecundity (eggs female^− 1^)	^a^ Fertility (% hatched larva		Female	Male		
Control	-	-	28.1 ± 2.1a (47)	96.8 ± 1.5a		18.3 ± 1.4a (32)	12.5 ± 0.9a (15)	-	-
Abamectin	3.6	36.17	6.5 ± 2.1b (13)	87.0 ± 8.3ab		18.0 ± 2.88a (13)	12.9 ± 1.5a (17)	86.82	III
Azadirachtin	24	47.94	9.0 ± 3.6b (8)	93.8 ± 6.3a		12.6 ± 2.27b (8)	7.1 ± 0.3c (15)	83.82	III
Chlorpyrifos	450	100.0	-	-		-	-	100	IV
Cypermethrin	62.5	100.0	-	-		-	-	100	IV
Deltamethrin	25	85.11	-	-		-	-	100	IV
Dimethoate	400	91.49	-	-		-	-	100	IV
Imidacloprid	100	51.43	3.3 ± 2.1c (9)	85.0 ± 9.57ab		13.6 ± 2.13b (9)	14.5 ± 3.19a (12)	94.95	III
Oxymatrine	2	38.53	7.9 ± 3.4b (8)	88.3 ± 3.33ab		14.3 ± 3.31b (8)	9.8 ± 1.24ab (18)	87.85	III
Propargite	360	82.98	17.6 ± 6.8b (5)	78.1 ± 7.7b		9.4 ± 5.6b (5)	8.0 ± 2.81b (3)	91.4	III
*Hirsutella* *thompsonii*	8	58.75	12.0 ± 4.1b (8)	90.0 ± 10.0a		10.8 ± 2.44b (8)	9.2 ± 1.51a b (11)	83.6	III

Means followed by the same letters in a column do not differ from each other. Standard errors (SE) were estimated using 100,000 bootstraps and means were compared using the paired bootstrap test at 5% significance

^a^Data (mean ± SE) followed by the same letter in a column do not differ significantly (GLM with quasi-binomial distribution, followed by post hoc Tukey test; *p* < 0.05)

^b^Reduction coefficient Ex of pesticides calculated by formula proposed by Biondi et al. (2012)

^c^ IOBC class toxicity in laboratory: I: harmless (Ex < 30%); II: slightly harmful (30% < Ex < 79%); III: moderately harmful (80% < Ex < 99%), and IV: highly harmful (Ex > 99%). The values in the brackets represent the number of replicates for each treatment

**Table 3:** Mean (± SE) life-table parameters of Phytoseiulus longipes when eggs were treated with pesticides

Treatment	Concentration used (mg i.a. L^− 1^)	*R*_0_ (Offspring/ individual)	*r*(day^− 1^)	λ (day^− 1^)	T (day)
Control	-	18.0 ± 2.32a	0.228 ± 0.011a	1.256 ± 0.013a	12.648 ± 0.294b
Abamectin	3.6	1.68 ± 0.65b	0.036 ± 0.03b	1.036 ± 0.031b	14.379 ± 3.059b
Azadirachtin	24	1.531 ± 0.75b	0.039 ± 0.069b	1.040 ± 0.065b	10.831 ± 0.729b
Imidacloprid	100	0.652 ± 0.43b	-0.033 ± 0.05c	0.967 ± 0.04c	12.966 ± 0.876b
Oxymatrine	2	1.4 ± 0.72b	0.028 ± 0.05b	1.029 ± 0.05b	11.762 ± 1.83b
Propargite	360	1.76 ± 1.54b	0.031 ± 0.10b	1.031 ± 0.09b	18.01 ± 4.20a
*Hirsutella thompsonii*	8	1.95 ± 0.88b	0.068 ± 0.05b	1.070 ± 0.05b	10.53 ± 0.84b

Means within a column followed by the same letter are not significantly different. The SEs were estimated by using 100,000 bootstraps and means were compared by using paired bootstrap test at 5% significance level. *R*_*0*_ = net reproductive rate; *r* = intrinsic rate of increase; *λ* = finite rate of increase; *T* = mean generation time

#### Age-specific survival rate and age-specific fecundity

**Fig. 2:**
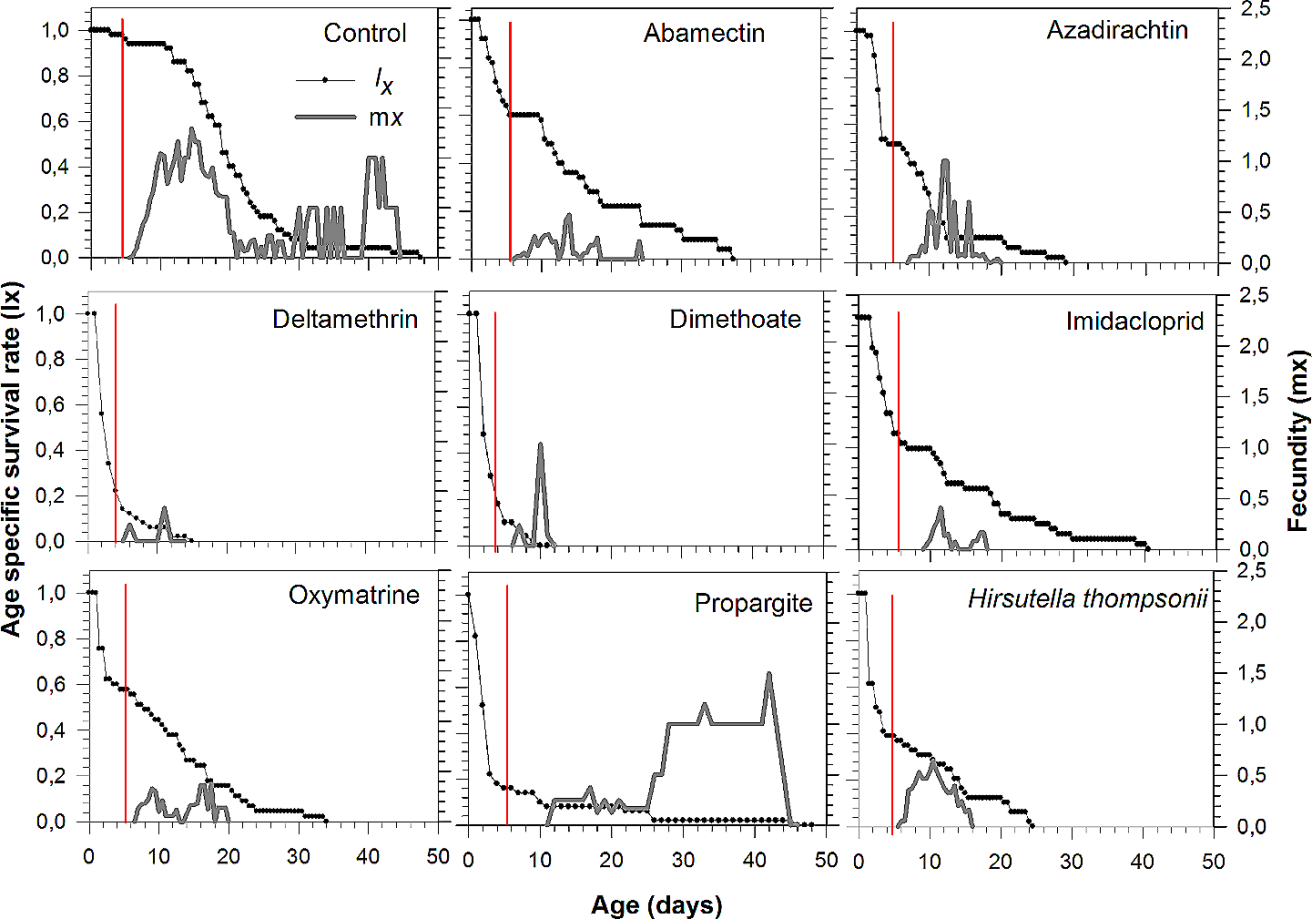
Age-specific survival rates (*l*_*x*_), age-specific fecundity (*m*_*x*_) of *Phytoseiulus longipes* when eggs were treated with pesticides. The lines in red in different treatments indicate the day of *P. longipes* adult emergence

Age-specific survival rate (*l*_x_) illustrates the likelihood of individual survival of *P. longipes* to age x (Fig. 2). Survival curves of *P. longipes* eggs exposed to pesticides clearly showed negative effects when compared to the control. Age range for 50% survivorship in the control (19.0 ± 0.91 days) was significantly longer than for pesticide-treated eggs: abamectin (11.0 ± 2.1), oxymatrine (7.5 ± 2.4), azadirachtin (6.5 ± 1.9), imidacloprid (5.0 ± 2.3), *H. thompsonii* (3.0 ± 0.9), propargite (1.6 ± 0.2), deltamethrin (1.5 ± 0.2), and dimethoate (1.2 ± 0.4). The life- span of *P. longipes* individuals from the control and propargite treatment was similar up to 46 days, which is significantly longer than the other treatments. The shortest life span was observed for mites from eggs exposed to dimethoate and deltamethrin. Age-specific fecundity (*m*_*x*_; average daily fecundity per individuals at age x) varied throughout the oviposition period (Fig. 2). Females from eggs exposed to imidacloprid and propargite started oviposition slightly later than those in control and other treatments. Compared to the control, *P. longipes* females from all other treatments stopped oviposition much earlier, except for propargite treatment, whose predators virtually oviposited until the end of their life span. The highest peak of specific daily fecundity was observed for females from eggs exposed to propargite. Maximum *m*_*x*_ values were 1.3 eggs (on the 15th day) for control, 1.2 eggs (on the 33rd day) for propargite treatment, and 1.0 eggs (on the 12th and 10th days) for azadirachtin and dimethoate treatments, respectively. In the other treatments, the maximum *m*_*x*_ value was less than 1.0 egg.

#### Lethal and sublethal effects on adults

All treatments showed significantly higher adult mortality compared to the control (F = 24.403; df = 10, 44; *p* < 0.01) (Table 4). Within 24 h, oxymatrine, chlorpyrifos, cypermethrin, deltamethrin, and dimethoate caused 88 to 100% adult mortality. Abamectin, imidacloprid, and propargite induced moderate mortality (52–82%), while azadirachtin and *H. thompsonii* resulted in lower mortality (36-37.5%). Females surviving azadirachtin, imidacloprid, and *H. thompsonii* treatments exhibited 2–3 times lower fecundity than control, while abamectin, oxymatrine, and propargite led to 5–11 times lower fecundity (F = 19.003; df = 6,69; *p* < 0.001). No significant differences in egg viability (fertility) were observed between the control and treatments with surviving females that oviposited (F = 1.089; df = 6,50; *p* = 0.38). Longevity of surviving adult females and males was shorter in all treatments compared to control (F = 24.039; df = 6,88; *p* < 0.001 and F = 16.149; df = 6,72; *p* < 0.001, respectively) (Table 4). Based on the reduction coefficient and IOBC/WPRS classification, chlorpyrifos, cypermethrin, deltamethrin, and dimethoate were highly harmful (class IV), while abamectin, imidacloprid, oxymatrine, and propargite were moderately harmful (class III) to *P. longipes* adults. Azadirachtin and *H. thompsonii* were classified as slightly harmful (class II).

**Table 4:** Lethal (adult mortality) within 24 h and sublethal effects (total fecundity, fertility, and female and male longevities), reduction coefficient and IOBC class toxicity of pesticides applied on adult Phytoseiulus longipes

Treatment	Dose used (mg i.a. L^− 1^)	Adult mortality^a^ (%)	Effect on reproduction (Er)			Longevity ^c^		Ex ^d^ (%)	IOBC/Class^e^
			Total fecundity^b^ (eggs female^− 1^)	Egg viability (%)		Female	Male		
Control	-	0.0 ± 0.0e	27.6 ± 1.91a	98.5 ± 1.47a		18.4 ± 1.2a	12.6 ± 0.93a	-	-
Abamectin	3.6	70.0 ± 5.48b	3.5 ± 0.83 cd	84.4 ± 8.10a		4.5 ± 0.34b	4.2 ± 0.31b	96.74	III
Azadirachtin	24	37.5 ± 5.48d	10.8 ± 5.2b	97.0 ± 5.0a		8.0 ± 2.16b	4.9 ± 0.91b	75.91	II
Chlorpyrifos	450	100.0 ± 2.00a	-	-		-	-	100	IV
Cypermethrin	62.5	100.0 ± 0.0a	-	-		-	-	100	IV
Deltamethrin	25	100.0 ± 0.0a	-	-		-	-	100	IV
Dimethoate	400	100.0 ± 0.0a	-	-		-	-	100	IV
Imidacloprid	100	68.0 ± 13.56b	8.3 ± 3.9b	93.8 ± 6.25a		5.6 ± 1.44b	6.1 ± 0.81b	90.84	III
Oxymatrine	2	82.0 ± 8.60a	2.5 ± 0.5d	87.5 ± 12.5a		4.0 ± 0.14b	3.7 ± 0.33b	98.6	III
Propargite	360	52.0 ± 3.74c	5.9 ± 2.3c	95.0 ± 3.62a		6.4 ± 1.26b	4.0 ± 0.97b	90.1	III
*Hirsutella* *thompsonii*	8	36.0 ± 13.26d	9.4 ± 2.4b	96.0 ± 6.12a		6.0 ± 0.99b	5.3 ± 0.8b	78.75	II
F		24.403	19.003	1.089		24.039	16.149		
d.f.		10,44	6,69	6,50		6,88	6,72		
P		< 0.001	< 0.001	0.3817		< 0.001	< 0.001		

^a^ Data (mean ± SE) followed by the same letter in a column do not differ significantly (GLM with quasi-binomial distribution, followed by post hoc Tukey test; *p* < 0.05)

^b^ Data (mean ± SE) followed by the same letter in a column do not differ significantly (GLM with quasi-Poisson distribution, followed by post hoc Tukey test; *p* < 0.05)

^c^ Data (mean ± SE) followed by the same letter in a column do not differ significantly (GLM with Gaussian distribution, followed by post hoc Tukey test; *p* < 0.05)

^d^ Reduction coefficient Ex of pesticides calculated by formula proposed by Biondi et al. (2012)

^e^ IOBC toxicity class in laboratory I: harmless (Ex < 30%); II: slightly harmful (30% < Ex < 79%); III: moderately harmful (80% < Ex < 99%), and IV: highly harmful (Ex > 99%)

#### Residual effects and duration of harmful activity of pesticides

Pesticide residue-ages significantly affect adult mortality and offspring production (Table 5). Interaction between residue-ages and treatments caused significant effect on adult mortality (χ2 = 41.03, df = 10; *p* < 0.001) and egg production of surviving females (χ2 = 116.91; df = 10; *p* < 0.001), but not for egg viability (χ2 = 96.5; df = 10; *p* = 0.64). For 4-day old residues, dimethoate, deltamethrin, cypermethrin and chlorpyrifos caused 100% adult mortality, while abamectin, propargite, oxymatrine and imidacloprid reduced mortality to 20–54% (F = 78.801, df = 10, 44; *p* < 0.001) (Table 5). Azadirachtin and *H. thompsonii* treatments resulted in mortality rates (8–10%) similar to control. Surviving females from azadirachtin treatment had egg production similar to control, while other treatments led to a 1.4–3.8-fold decrease in fecundity (F = 5 7.072; df = 10, 44; *p* < 0.001).

**Table 5:** Mean (± SE) of mortality and reproductive parameters of adult Phytoseiulus longipes, reduction coefficient and IOBC class toxicity of pesticides applied after 72-h exposure to 4, 10, 20 and 31 DAA aged residues of pesticides under screen-house (26.7 ± 0.3 oC, RH 59 ± 1%, ca. 10 h of daily light)

Treatment	Concentration used (mg i.a. L^− 1^)	Adult mortality^a^	Effect on reproduction (Er)		Ex (%)	IOBC Class ^e^		Adult mortality^a^ (M_c_ %)	Effect on reproduction (Er)		Ex (%)	IOBC Class ^e^
			Number of eggs/ live females^b^	% of egg viability^a^					Number of eggs/ live females^b^	% of egg viability^a^		
**4DAA**								**10 DAA**				
Control	-	2.7 ± 1.4c	38.4 ± 1.5a	100.0 ± 0.0a	-	-		3.1 ± 1.1c	39.2 ± 2.2a	100.0 ± 0.0a	-	-
Abamectin	3.6	38.0 ± 6.6b	23.4 ± 1.9b	70.1 ± 8.1b	72.45	III		4.0 ± 2.4c	31.6 ± 2.6ab	98.0 ± 6.8b	24.2	I
Azadirachtin	24	8.1 ± 2.0c	35.0 ± 3.5a	94.0 ± 2.5a	17.89	I						
Chlorpyrifos	450	100.0 ± 0.0a	0.0 ± 0.0d	-	100	IV		98.0 ± 1.3a	2.2 ± 1.2d	6.0 ± 2.4c	100	IV
Cypermethrin	62.5	100.0 ± 0.0a	0.0 ± 0.0d	-	100	IV		94.0 ± 2.5a	2.4 ± 0.6d	24.0 ± 6.8c	100	IV
Deltamethrin	25	100.0 ± 0.0a	0.0 ± 0.0d	-	100	IV		98.0 ± 0.0a	1.1 ± 1.1d	22.0 ± 4.9c	100	IV
Dimethoate	400	98.0.0 ± 0.0a	0.0 ± 0.0d	-	100	IV		97.0 ± 0.0a	1.5 ± 1.2d	6.0 ± 2.4c	100	IV
Imidacloprid	100	36.2 ± 2.3b	23.2 ± 3.5b	72.0 ± 3.7b	70.99	III		14.0 ± 8.7bc	23.6 ± 3.5bc	78.0 ± 4.9b	57.9	III
Oxymatrine	2	20.1 ± 4.5b	27.8 ± 2.6b	92.0 ± 3.8a	44.5	II		8.0 ± 3.7c	36.4 ± 3.3a	94.0 ± 2.5ab	16.4	I
Propargite	360	54.0 ± 9.3b	10.0 ± 1.9c	80.0 ± 6.3b	90.1	III		30.0 ± 7.7b	16.0 ± 2.7c	86.0 ± 4.1b	74.4	III
*Hirsutella thompsonii*	8	10.0 ± 3.2c	26.6 ± 2.2b	94.0 ± 2.5a	39.0	II		4.3 ± 2.2c	37.8 ± 1.1a	96.0 ± 2.5a	7.4	I
F (d.f)		78.8(10,44)	57.1(10,44)	13.1(6,28)				98.7(9,40)	63.1(9,40)	78.8(9, 40)		
P		< 0.001	< 0.001	< 0.001				< 0.001	< 0.001	< 0.001		
**20 DAA**								**31 DAA**				
Control	-	2.1 ± 1.1b	37.3 ± 2.2a	98.1 ± 1.2a	-	-		2.3 ± 1.4b	35.2 ± 2.2a	98.6 ± 0.9a	-	-
Chlorpyrifos	450	86.0 ± 8.7a	3.6 ± 0.2b	16.0 ± 4.0b	99.8	IV		57.0 ± 3.7a	7.6 ± 1.4b	49.8 ± 6.0b	95.3	IV
Cypermethrin	62.5	82.0 ± 9.7a	4.6 ± 1.5b	36.0 ± 5.1b	99.2	IV		67.0.0 ± 2.8a	8.4 ± 0.6b	64.6 ± 6.2b	94.8	IV
Deltamethrin	25	78.0 ± 10.2a	5.4 ± 0.6b	39.0 ± 5.6b	98.7	IV		56.4 ± 4.9a	7.6 ± 0.2b	58.4 ± 3.1b	94.4	IV
Dimethoate	400	70.0 ± 9.4a	6.6 ± 1.2b	28.0 ± 11.1b	98.5	IV		54.8 ± 4.8a	10.5 ± 1.3b	54.0 ± 7.2b	92.6	IV
Imidacloprid	100	6.0 ± 4.9b	33.5 ± 3.4a	97.0 ± 4.0a	16.5	I						
Propargite	360	8.0 ± 3.5b	34.8 ± 2.3a	98.0 ± 1.2a	14.3	I						
F (d.f)		29.6(6,28)	76.7(6,28)	47.1(6,28)				53.8(4,20)	60.1(4,20)	56.3(4,20)		
P		< 0.001	< 0.001	< 0.001				< 0.001	< 0.001	< 0.001		

Data followed by the same letter and for the same age residue in a column do not differ significantly.

^a^(GLM with quasi-binomial distribution, followed by post hoc Tukey test; *p* < 0.05)

^b^(GLM with quasi-Poisson distribution, followed by post hoc Tukey test; *p* < 0.05)

^c^ (GLM with Gaussian distribution, followed by post hoc Tukey test; *p* < 0.05)

^d^Ex= 100-(100-Mc) x R1xR2

^e^ IOBC toxicity class used in extended laboratory test: I: harmless (Ex < 25%); II: slightly harmful (25% < Ex < 50%); III: moderately harmful (51% < Ex < 75%), and IV: highly harmful (Ex > 75%)

Egg viability was reduced to 70–80% in abamectin, propargite and imidacloprid treatments, with no significant effect observed for other treatments compared to the control (F = 13.129; df = 6,28; *p* < 0.001). For 10-day old residues, *H. thompsonii*, abamectin, oxymatrine and imidacloprid had effects statistically similar to control, with propargite reducing mortality to 24%. For other treatments, adult mortality remained high (~ 94–98%) (F = 98.1, df = 9, 40; *p* < 0.001). Surviving females exposed to *H. thompsonii*, oxymatrine and abamectin had egg production similar to the control, while propargite and imidacloprid treatments resulted in 1.5–2.5 times lower fecundity (F = 63.1; df = 9,40; *p* < 0.001). Egg viability in chlorpyrifos, cypermethrin, deltamethrin and dimethoate treatments was 6–24%, while other treatments followed a similar trend as reported for 4-day old residues, except for imidacloprid, which did not differ from the control (F = 78.8; df = 9,40; *p* < 0.001). For 20-day old residues, dimethoate, deltamethrin, cypermethrin and chlorpyrifos caused 70–86% adult mortality (F = 29.6, df = 6,28; *p* < 0.001), while imidacloprid, abamectin and propargite treatments had no significant effect on adult mortality, ranging between 4 and 8%. Surviving females exposed to imidacloprid and propargite had egg production statistically similar to control, while other treatments led to a substantial reduction in fecundity (F = 76.7; df = 6,28; *p* < 0.001). Egg viability followed the inverse trend reported for adult mortality (F = 47.1; df = 6,28; *p* < 0.001). For 31-day old residues, mortality due to dimethoate, deltamethrin, cypermethrin, or chlorpyrifos treatments was 54–67% higher than the control (F = 53.8; df = 4,20; *p* < 0.001). Number of eggs produced by surviving females (F = 60.1; df = 4,20; *p* < 0.001) and egg viability (F = 56.3; df = 4,20; *p* < 0.001) followed the inverse trend reported for adult mortality.

Based on the reduction coefficient (Ex) and the IOBC classification, azadirachtin was classified as harmless for 4-day old residue (Table 5). Oxymatrine and *H. thompsonii* were initially slightly harmful at 4-day residue but became harmless at 10-day residue, while abamectin was observed as moderately harmful at 4-day residue and turned harmless at 10-day residue. Propargite and imidacloprid, initially moderately harmful at 4- and 10-day residue respectively, became harmless at 20-day residue. Other pesticides (chlorpyrifos, cypermethrin, deltamethrin, dimethoate) remained highly harmful across all residue ages. According to the IOBC persistence toxicity, azadirachtin was classified as short-lived (A: <5 days), and abamectin, oxymatrine, and *H. thompsonii* were slightly persistent (B: 5–15 days). Propargite and imidacloprid were moderately persistent (C: 16–30 days), while other pesticides (chlorpyrifos, cypermethrin, deltamethrin, dimethoate) were highly persistent (D: >30 days).

## Discussion

In this study, we thoroughly examined the lethal and sublethal effects of ten synthetic and biological pesticides at their maximum field-recommended concentration on the predatory mite *P. longipes* in both egg and adult stages. The results showed contrasting non-target effects depending on the life stage, chemical group, and/or exposure route and residue age. For instance, chlorpyrifos and cypermethrin, when applied topically, were found to be acutely toxic to both egg and adult stages, resulting survival rates in 0-6.1%. In contrast, abamectin, deltamethrin, dimethoate, oxymatrine, and imidacloprid caused higher toxicity to adults (68–100%) compared to eggs (2.0-15.6%), but all showed significant sublethal effects on the developmental time, fecundity, fertility, or longevity of surviving *P. longipes* stages, with the effect of deltamethrin and dimethoate being apparent. Azadirachtin, propargite, and *H. thompsonii*, despite having lower and moderate acute toxicity rates for both eggs (12.8–22%) and adults (36–52%) demonstrated a significant effect on development and reproduction of surviving *P. longipes* stages. These results are comparable to what has been reported in previous studies on other phytoseid species. Interestingly, we also observed that pesticides highly harmful to *P. longipes* through topical exposure in laboratory conditions are not necessarily persistent under screen-house conditions. These findings highlight the importance of assessing non-target effects on each predator life stage and exposure route (Duso et al. 2008; Biondi et al. 2013; Put et al. 2016; Fernández et al. 2017; Franco et al. 2017; Zanardi 2017; Bergeron and Schmidt-Jeffris 2020; Döker and Kazak 2020).

The reduction coefficient (Ex) provides a comprehensive measure of risk, considering both acute toxicity and sublethal effects of pesticides on biological control compatibility (Biondi et al. 2012). In our study, using this approach and IOBC classification (Hassan et al. 1994; Biondi et al. 2012), we found that the topical exposure of two organophosphates- chlorpyrifos and dimethoate, as well as two pyrethroids- cypermethrin and deltamethrin, was highly harmful (IOBC Class IV) to both *P. longipes* eggs and adult stages. Even 31 days after spraying, these pesticides continue to exert a highly harmful residual activity on *P. longipes* adults. Previous studies consistently reported similar harmful effects and persistence of pyrethroids and organophosphates on various natural enemies, including phytoseiid species (Abou-Awad and El-Banhawy 1985; Villanueva and Walgenbach 2005; Broufas 2008; Bostanian et al. 2010; Hamby et al. 2013; Beers and Schmidt 2014; Franco et al. 2017; Schmidt-Jeffris et al. 2021). These results suggest that organophosphates and pyrethroids are not compatible with *P. longipes*. Therefore, implementing IPM programs involving *P. longipes* in areas where these pesticides are extensively used may lead to inadequate pest control.

Direct exposure to imidacloprid, abamectin and propargite had moderately harmful effects (IOBC Class III) on both eggs and adults. This harm was caused by either high acute toxicity or strong sublethal effects on post-embryonic development and reproduction parameters in the tested egg and adult stages. Other studies have also demonstrated such effects of imidacloprid on the demographic parameters of various phytoseiid mites, including *Neoseiulus californicus* McGregor (Villanueva and Walgenbach 2005; Argolo PS 2013), *Euseius gallicus* Kreiter & Tixier and *Phytoseiulus persimilis* (Athias-Henriot) (Put et al. 2016) as well as *Amblyseius andersoni* (Chant), *Galendromus occidentalis* (Nesbitt) and *Neoseiulus fallacis* (Garman) (James 2003). Similarly, high acute toxicity or dramatic sublethal effects were reported for abamectin or propargite against phytoseiids such as *E. gallicus*, *Euseius scutalis* Athias-Henriot, *N. californicus*, *N. fallacis*, *P. macropilis*, *P. persimilis* and *Typhlodromus pyri* Scheuten (Hardman et al. 2003; Cote et al. 2004; Bostanian and Akalach 2006; Put et al. 2016; Döker and Kazak 2020). Moreover, the intrinsic rate of increase (*r*), a life table parameter reflecting the combined effects of biological attributes such as survival, sex ratio, developmental duration and fecundity (Janssen and Sabelis 1992), was found to be negative (-0.03 ± 0.06 day^− 1^) for *P. longipes* exposed to imidacloprid and substantially lower for abamectin (0.04 ± 0.03) and propargite (0.03 ± 0.1). These results suggest that topical exposure to abamectin and propargite could reduce the population of *P. longipes*, potentially hindering its effectiveness as a biological control agent of *T. evansi*. In contrast, imidacloprid could lead to predator suppression over time.

Certain pesticides, initially considered incompatible with biological control through topical exposure, may actually be compatible when it comes to residual exposure (Fernández et al. 2017). However, the length of time necessary for an applied pesticide to become innocuous can vary significantly, depending on the predator species and pesticide group (Wanumen et al. 2016; Franco et al. 2017). For instance, previous studies have shown that residues propargite became harmless to *N. californicus* 16–30 days post-spraying, suggesting moderate persistence (Uddin et al. 2015). Conversely, residues of abamectin turned innocuous to *Amblyseius swirskii* Athias-Henriot, *N. californicus* and *P. persimilis* 5–15 days after spraying, suggesting their slight persistence (Van de Veire et al. 2002; Sáenz-de-Cabezón Irigaray et al. 2007; Ruiz and de Moraes 2008; Nadimi et al. 2011; Uddin et al. 2015; Fernández et al. 2017). On the other hand, Franco et al. (2017) and Zanardi (2017) observed harmful effects of imidacloprid on *Euseius concordis* (Cant) and *Iphiseiodes zuluagai* Denmark & Muma lasted only three days in citrus, whereas Wanumen et al. (2016) reported 34 days of harmful effects on *Macrolophus basicornis* (Stal) (Heteroptera: Miridae). In line with these findings, our study revealed that the residues of abamectin and oxymatrine remained harmful to *P. longipes* for 5–15 days post-spraying (IOBC class B, slight persistence), while that of propargite and imidacloprid turned non-toxic to *P. longipes* 16–30 days after application (IOBC class C, moderate persistence).

Azadirachtin, oxymatrine, and the pathogenic fungus *H. thompsonii*, are well-known environmentally-friendly pesticides used in crop pest management (Chandler et al. 2005; Biondi et al. 2013; de Andrade et al. 2019). However, in this study, we found that their selectivity towards *P. longipes* varied greatly. For instance, topical exposure to oxymatrine significantly reduced the intrinsic rate of increase (*r*: 0.03 ± 0.05 day^− 1^) and caused moderate harm to both egg and adult stages (IOBC class III), but it became harmless to *P. longipes* 10 days post-application (slight persistence). Conversely, topical exposure to azadirachtin and *H. thompsonni* showed slight harm to adult stage (IOBC Class II), but moderate harm to eggs (IOBC Class III), lowering the *r*-value (*r*: 0.04 ± 0.07 and 0.07 ± 0.05 day^− 1^ respectively). Furthermore, the harmful residual activity of azadirachtin lasted less than 5 days (short persistence, IOBC A), whereas the residual persistence of *H. thompsonii* was similar to that of oxymatrine. These findings suggest that while oxymatrine may not be suitable for conserving the *P. longipes* population, as observed in our previous study (Savi et al. 2021b), its low persistence may allow the use in well-timed augmentative releases. Conversely, azadirachtin and *H. thompsonii* may be partially adequate for use in a conservation strategy of biological control, considering their relatively short persistence on *P. longipes*.

Oxymatrine is known for its strong acaricidal activity which targets nicotinic acetylcholine receptors and sodium channels in arthropod nerve cells (Ali et al. 2017; de Andrade et al. 2019), resulting in high toxicity in adults and significant adverse effects on fecundity and longevity. This may explain the observed reduced intrinsic rate of increase or moderate harm in both egg and adult stages following topical exposure in our study. These results are consistent with those of Shah and Appleby (2019) who reported high acute toxicity and dramatic sublethal effects on fecundity in *N. fallacis*, *P. persimilis* and *Stethorus punctillum* Weise (Coleoptera: Coccinellidae) after topical exposure to oxymatrine. In contrast, Fang et al. (2018) found low mortality of *Neoseiulus cucumeris* (Oudemans) females exposed to oxymatrine residual contact. de Andrade et al. (2019) also found no impact on population levels of phytoseiids, including *Amblyseius chiapensis* De Leon, *Amblyseius* sp., and *I. zuluagai* on citrus following their contact with residues of this pesticide. The difference in results can be attributed to variations in experimental conditions, such as dosage, exposure duration, and application methods, as well as the sensitivity of the species tested. Regarding the compatibility between *H. thomposonni* and other bio control agents, there are no studies available. However, research on other entomopathogenic fungi, such as *Beauveria bassiana* (Balsamo) (Cordycipitaceae) or *Isaria fumosorosea* (Wize) Brown & Smith (Cordycipitaceae) has reported adverse effects on the survival, longevity, and fecundity of phytoseiid mites (Ullah and Lim 2017; Zemek et al. 2017). In contrast to our results, Wekesa et al. (2007) reported that the fecundity of *P. longipes* was not affected when feeding on *T. evansi* or *T. urticae* infected with *Neozygites floridana* (Weiser & Muma) (Neozygitaceae). In our study, the prey offered to *P. longipes* was not infected by the fungus *H. thompsonii*, but it could have been chemically affected by the insecticides used in the different treatments.

Azadirachtin is known for disrupting arthropod molting by interfering with ecdysone synthesis, resulting in extended developmental time and reduced reproductive capabilities (Mordue and Blackwell 1993; Biondi et al. 2012, 2013). This aligns with our observation of high sensitivity of *P. longipes* eggs over the adult stage in this study and, consistently with findings from similar studies involving *N. cucumeris* and *P. persimilis* (Spollen and Isman 1996), *N. californicus* and *P. macropilis* (Bernardi et al. 2013), and *Neoseiulus barkeri* (Athias-Henriot) (Silva et al. 2023). Short-lived persistence of residual effect of azadirachtin has been reported on *E. gallicus* and *Euseius stipulatus* Athias-Henriot (Viggiani and Bernardo 2001; Put et al. 2016).

## Conclusion

In summary, our study demonstrated significant variations in the lethal and sublethal effects of ten synthetic biological pesticides on both egg and adult stages of *P. longipes*. Through topical exposure assessment, pyrethroid and organophosphate pesticides were found to be highly harmful to both stages. On the other hand, oxymatrine, imidacloprid, abamectin, and propargite were determined to be moderately harmful. Azadirachtin and *H. thompsonni* were slightly harmful to adults, but azadirachtin was found to be harmless after 4-days of the application. Similarly, abamectin, oxymatrine, and *H. thompsonii* and propargite were considered harmless after respectively 10 and 20 days of application. Pyrethroids and organophosphates remained highly harmful for up to 31-days, suggesting these products not to be appropriate for IPM programs that involve the introduction of *P. longipes*. Conversely, azadirachtin should be considered the safest option. In summary, care should be taken in relation to safety deadlines before releasing that predatory mite. These crucial insights will aid in the integration of *P. longipes* into IPM aimed at controlling *T. evansi*. Future studies should include field trials to validate the findings of this work.

## Data Availability

No datasets were generated or analysed during the current study.

## References

[CR1] Abad-Moyano R, Pina T, Ferragut F, Urbaneja A (2009) Comparative life-history traits of three phytoseiid mites associated with *Tetranychus urticae* (Acari: Tetranychidae) colonies in clementine orchards in eastern Spain: implications for biological control. Exp Appl Acarol 47:121–132. 10.1007/s10493-008-9197-z 18931925 10.1007/s10493-008-9197-z

[CR2] Abou-Awad BA, El-Banhawy EM (1985) Comparison between the toxicity of synthetic pyrethroids and other compounds to the predacious mite *Amblyseius Gossipi* (Mesostigmata: Phytoseiidae). Exp Appl Acarol 1:185–191. 10.1007/BF01198515 10.1007/BF01198515

[CR72] Agrofit (2021) Sistema de Agrotóxicos Fitossanitários - Ministério da Agricultura, Pecuária e Abastecimento, Brasil. In. http://extranet.agricultura.gov.br/agrofit_cons/principal_agrofit_cons. Accessed Accessed 14 Jan 2021

[CR3] Ali S, Zhang C, Wang Z, Wang XM, Wu JH, Cuthbertson AGS, Shao Z, Qiu BL (2017) Toxicological and biochemical basis of synergism between the entomopathogenic fungus *lecanicillium muscarium* and the insecticide matrine against *Bemisia tabaci* (Gennadius). Sci Rep 7:46558. 10.1038/srep46558 28425450 10.1038/srep46558 PMC5397844

[CR4] Argolo PSBN, Santiago S, Mollá Ó, Jacas JA, Urbaneja A (2013) Compatibility of *Phytoseiulus Persimilis* and *Neoseiulus californicus* (Acari: Phytoseiidae) with imidacloprid to manage clementine nursery pests. Crop Prot 43:175–182. 10.1016/j.cropro.2012.09.018 10.1016/j.cropro.2012.09.018

[CR5] Azandémè-Hounmalon GY, Affognon HD, Komlan FA, Tamò M, Fiaboe KKM, Kreiter S, Martin T (2015) Farmers’ control practices against the invasive red spider mite, *Tetranychus Evansi* Baker & Pritchard in Benin. Crop Prot 76:53–58. 10.1016/j.cropro.2015.06.007 10.1016/j.cropro.2015.06.007

[CR6] Beers EH, Schmidt RA (2014) Impacts of orchard pesticides on *Galendromus Occidentalis*: Lethal and sublethal effects. Crop Prot 56:16–24. 10.1016/j.cropro.2013.10.010 10.1016/j.cropro.2013.10.010

[CR7] Bergeron PE, Schmidt-Jeffris RA (2020) Not all predators are equal: miticide non-target effects and differential selectivity. Pest Manag Sci 76:2170–2179. 10.1002/ps.5754 31955529 10.1002/ps.5754

[CR8] Bernardi D, Botton M, da Cunha US, Bernardi O, Malausa T, Garcia MS, Nava DE (2013) Effects of azadirachtin on *Tetranychus urticae* (Acari: Tetranychidae) and its compatibility with predatory mites (Acari: Phytoseiidae) on strawberry. Pest Manag Sci 69:75–80. 10.1002/ps.3364 22807305 10.1002/ps.3364

[CR11] Biondi A, Campolo O, Desneux N, Siscaro G, Palmeri V, Zappalà L (2015) Life stage-dependent susceptibility of *Aphytis Melinus* DeBach (Hymenoptera: Aphelinidae) to two pesticides commonly used in citrus orchards. Chemosphere 128:142–147. 10.1016/j.chemosphere.2015.01.034 25698292 10.1016/j.chemosphere.2015.01.034

[CR9] Biondi A, Desneux N, Siscaro G, Zappalà L (2012) Using organic-certified rather than synthetic pesticides may not be safer for biological control agents: selectivity and side effects of 14 pesticides on the predator *Orius Laevigatus*. Chemosphere 87:803–812. 10.1016/j.chemosphere.2011.12.082 22342338 10.1016/j.chemosphere.2011.12.082

[CR10] Biondi A, Zappalà L, Stark JD, Desneux N (2013) Do Biopesticides affect the demographic traits of a parasitoid wasp and its Biocontrol Services through Sublethal effects? PLoS ONE 8 10.1371/journal.pone.0076548 PMC3787011 24098793

[CR12] Bostanian NJ, Akalach M (2006) The effect of indoxacarb and five other insecticides on *Phytoseiulus Persimilis* (Acari: Phytoseiidae), *Amblyseius fallacis* (Acari: Phytoseiidae) and nymphs of *Orius insidiosus* (Hemiptera: Anthocoridae). Pest Manag Sci 62:334–339. 10.1002/ps.1171 16493722 10.1002/ps.1171

[CR13] Bostanian NJ, Hardman JM, Thistlewood HA, Racette G (2010) Effects of six selected orchard insecticides on *Neoseiulus fallacis* (Acari: Phytoseiidae) in the laboratory. Pest Manag Sci 66:1263–1267. 10.1002/ps.2010 20715016 10.1002/ps.2010

[CR14] Broufas GD, Pappas M, Vassiliou G, Koveos DS (2008) Toxicity of certain pesticides to the predatory mite *Euseius finlandicus* (Acari: Phytoseiidae). IOBC WPRS Bull 85:85–91

[CR15] Chandler D, Davidson G, Jacobson RJ (2005) Laboratory and glasshouse evaluation of entomopathogenic fungi against the two-spotted spider mite, *Tetranychus urticae* (Acari: Tetranychidae), on tomato, *Lycopersicon esculentum*. Biocontrol Sci Technol 15:37–54. 10.1080/09583150410001720617 10.1080/09583150410001720617

[CR16] Chi H (2022) TWOSEX-MSChart: a computer program for the age-stage, two-sex life table analysis. In. http://140.120.197.173/Ecology/prod02.htm Accessed June 2022 2022

[CR17] Cote KW, Schultz PB, Lewis EE (2004) Using Acaricides in Combination with *Phytoseiulus Persimilis* Athias-Henroit to suppress *Tetranychus Urticae* Koch populations. J Entomol 39:267–274. 10.18474/0749-8004-39.2.267 10.18474/0749-8004-39.2.267

[CR18] de Andrade DJ, Ribeiro EB, de Morais MR, Zanardi OZ (2019) Bioactivity of an oxymatrine-based commercial formulation against *Brevipalpus Yothersi* Baker and its effects on predatory mites in citrus groves. Ecotoxicol Environ Saf 176:339–345. 10.1016/j.ecoenv.2019.03.118 30953999 10.1016/j.ecoenv.2019.03.118

[CR19] de Moraes G, McMurtry J, Denmark H, Campos C (2004) A revised catalog of the mite family Phytoseiidae. Zootaxa 434:1–494 10.11646/zootaxa.434.1.1

[CR20] Desneux N, Decourtye A, Delpuech JM (2007) The sublethal effects of pesticides on beneficial arthropods. Annu Rev Entomol 52:81–106. 10.1146/annurev.ento.52.110405.091440 16842032 10.1146/annurev.ento.52.110405.091440

[CR21] Döker İ, Kazak C (2020) Toxicity and risk assessment of acaricides on the predatory mite, *Euseius Scutalis* (Athias-Henriot) (Acari: Phytoseiidae) under laboratory conditions. Chemosphere 261:127760. 10.1016/j.chemosphere.2020.127760 32731029 10.1016/j.chemosphere.2020.127760

[CR22] Duso C, Malagnini V, Pozzebon A, Castagnoli M, Liguori M, Simoni S (2008) Comparative toxicity of botanical and reduced-risk insecticides to Mediterranean populations of *Tetranychus urticae* and *Phytoseiulus Persimilis* (Acari Tetranychidae, Phytoseiidae). Biol Control 47:16–21. 10.1016/j.biocontrol.2008.06.011 10.1016/j.biocontrol.2008.06.011

[CR23] Duso C, Van Leeuwen T, Pozzebon A (2020) Improving the compatibility of pesticides and predatory mites: recent findings on physiological and ecological selectivity. Curr Opin Insect Sci 39:63–68. 10.1016/j.cois.2020.03.005 32330876 10.1016/j.cois.2020.03.005

[CR24] Efron B, Tibshirani R (1994) An introduction to the bootstrap, monographs on statistics and applied probability. New York, Chapman and Hall/CRC

[CR25] Fang XD, Ouyang GC, Lu HL, Guo MF, Wu WN (2018) Ecological control of citrus pests primarily using predatory mites and the bio-rational pesticide matrine. Int J Pest Manag 64:262–270. 10.1080/09670874.2017.1394507 10.1080/09670874.2017.1394507

[CR26] Fernández MM, Medina P, Wanumen A, Del Estal P, Smagghe G, Viñuela E (2017) Compatibility of sulfoxaflor and other modern pesticides with adults of the predatory mite *Amblyseius Swirskii*. Residual contact and persistence studies. Biocontrol 62:197–208. 10.1007/s10526-017-9784-1 10.1007/s10526-017-9784-1

[CR28] Ferrero M, Calvo FJ, Atuahiva T, Tixier MS, Kreiter S (2011) Biological control of Tetranychus Evansi Baker & Pritchard and Tetranychus Urticae Koch by Phytoseiulus Longipes Evans in tomato greenhouses in Spain [Acari: Tetranychidae, Phytoseiidae]. Biol Control 58:30–35. 10.1016/j.biocontrol.2011.03.012 10.1016/j.biocontrol.2011.03.012

[CR27] Ferrero M, de Moraes GJ, Kreiter S, Tixier MS, Knapp M (2007) Life tables of the predatory mite *Phytoseiulus longipes* feeding on *Tetranychus evansi* at four temperatures (Acari: Phytoseiidae, Tetranychidae). Exp Appl Acarol 41:45–53. 10.1007/s10493-007-9053-6 17334816 10.1007/s10493-007-9053-6

[CR29] Franco AA, Zanardi OZ, Jacob CRO, de Oliveira MBR, Yamamoto PT (2017) Susceptibility of *Euseius Concordis* (Mesostigmata: Phytoseiidae) to pesticides used in citrus production systems. Exp Appl Acarol 73:61–77. 10.1007/s10493-017-0176-0 28866797 10.1007/s10493-017-0176-0

[CR30] Hamby KA, Alifano JA, Zalom FG (2013) Total effects of contact and residual exposure of bifenthrin and λ-cyhalothrin on the predatory mite *Galendromus Occidentalis* (Acari: Phytoseiidae). Exp Appl Acarol 61:183–193. 10.1007/s10493-013-9680-z 23446744 10.1007/s10493-013-9680-z

[CR31] Hardman JM, Franklin JL, Moreau DL, Bostanian NJ (2003) An index for selective toxicity of miticides to phytophagous mites and their predators based on orchard trials. Pest Manag Sci 59:1321–1332. 10.1002/ps.769 14667054 10.1002/ps.769

[CR32] Hassan SA, Bigler F, Bogenschütz H, Boller E, Brun J, Calis JNM, Coremans-Pelseneer J, Duso C, Grove A, Heimbach U, Helyer N, Hokkanen H, Lewis GB, Mansour F, Moreth L, Polgar L, Samsøe-Petersen L, Sauphanor B, Stäubli A, Sterk G, Vainio A, van de Veire M, Viggiani G, Vogt H (1994) Results of the sixth joint pesticide testing programme of the IOBC/WPRS-working group «pesticides and beneficial organisms». Entomophaga 39:107–119. 10.1007/BF02373500 10.1007/BF02373500

[CR33] Henderson CF, Tilton EW (1955) Tests with acaricides against the brown wheat mite. J Econ Entomol 48:157–161 10.1093/jee/48.2.157

[CR34] Hinde J, Demétrio CGB (1998) Overdispersion: models and estimation. Comput Stat Data Anal 27:151–170. 10.1016/S0167-9473(98)00007-3 10.1016/S0167-9473(98)00007-3

[CR35] James DG (2003) Toxicity of imidacloprid to *Galendromus Occidentalis*, *Neoseiulus fallacis* and *Amblyseius andersoni* (Acari: Phytoseiidae) from hops in Washington State. USA Exp Appl Acarol 31:275–281. 10.1023/b:appa.0000010383.33351.2f 14974692 10.1023/b:appa.0000010383.33351.2f

[CR36] Janssen A, Sabelis MW (1992) Phytoseiid life-histories, local predator-prey dynamics, and strategies for control of tetranychid mites. Exp Appl Acarol 14:233–250. 10.1007/BF01200566 10.1007/BF01200566

[CR37] Kim SY, Ahn HG, Ha PJ, Lim UT, Lee J-H (2018) Toxicities of 26 pesticides against 10 biological control species. J Asia Pac Entomol 21:1–8. 10.1016/j.aspen.2017.10.015 10.1016/j.aspen.2017.10.015

[CR38] Knapp M, Van Houten Y, Van Baal E, Groot T (2018) Use of predatory mites in commercial biocontrol: current status and future prospects. Acarologia 58:72–82. 10.24349/acarologia/20184275 10.24349/acarologia/20184275

[CR39] McMurtry J, de Moraes GJ, Sourassou NF (2013) Revision of the lifestyles of phytoseiid mites (Acari:Phytoseiidae) and implications for biological control strategies. Syst Appl Acarol 18:297–320. 10.11158/saa.18.4.1 10.11158/saa.18.4.1

[CR40] Mordue AJ, Blackwell A (1993) Azadirachtin: an update. J Insect Physiol 39:903–924. 10.1016/0022-1910(93)90001-8 10.1016/0022-1910(93)90001-8

[CR41] Mordue AJ, Nisbet AJ (2000) Azadirachtin from the neem tree Azadirachta indica: its action against insects, vol 29. Anais da Sociedade Entomológica do Brasil

[CR42] Nadimi A, Kamali K, Arbabi M, Abdoli F (2011) Study on persistence tests of miticides abamectin and fenproximate to predatory mite *Phytoseiulus Persimilis* (Acarina: Phytoseiidae). Afr J Agric Res 6:338–342

[CR43] Navajas M, de Moraes GJ, Auger P, Migeon A (2013) Review of the invasion of *Tetranychus Evansi*: biology, colonization pathways, potential expansion and prospects for biological control. Exp Appl Acarol 59:43–65. 10.1007/s10493-012-9590-5 22824945 10.1007/s10493-012-9590-5

[CR44] Nelder JA, Wedderburn RWM (1972) Generalized Linear models. J R Stat Soc Ser Stat Soc 135:370–384. 10.2307/2344614 10.2307/2344614

[CR45] Put K, Bollens T, Wäckers F, Pekas A (2016) Non-target effects of commonly used plant protection products in roses on the predatory mite *Euseius Gallicus* Kreiter & Tixier (Acari: Phytoseidae). Pest Manag Sci 72:1373–1380. 10.1002/ps.4162 26434923 10.1002/ps.4162

[CR46] Ruiz MG, de Moraes GJ (2008) Mortalidade do ácaro predador *Neoseiulus californicus* (Acari: Phytoseiidae) em testes de toxicidade residual de inseticidas e acaricidas usuais em pomáceas. Rev Bras Frutic 30:919–924. 10.1590/S0100-29452008000400014 10.1590/S0100-29452008000400014

[CR48] Sato Y, Mochizuki M, Mochizuki A (2012) Introduction of non-native predatory mites for Pest Control and its Risk Assessment in Japan. Jpn Agric Res Q 46:129–137. 10.6090/jarq.46.129 10.6090/jarq.46.129

[CR50] Savi PJ, de Moraes GJ, de Andrade DJ (2021a) Effect of tomato genotypes with varying levels of susceptibility to *Tetranychus evansi* on performance and predation capacity of *Phytoseiulus longipes*. Biocontrol 66:687–700. 10.1007/s10526-021-10096-5 10.1007/s10526-021-10096-5

[CR49] Savi PJ, de Moraes GJ, Melville CC, Andrade DJ (2019) Population performance of *Tetranychus evansi* (Acari: Tetranychidae) on African tomato varieties and wild tomato genotypes. Exp Appl Acarol 77:555–570. 10.1007/s10493-019-00364-6 31055676 10.1007/s10493-019-00364-6

[CR51] Savi PJ, Martins MB, de Moraes GJ, Cossi Hountondji FC, Andrade DJ (2021b) Bioactivity of oxymatrine and azadirachtin against *Tetranychus evans*i (Acari: Tetranychidae) and their compatibility with the predator *Phytoseiulus longipes* (Acari: Phytoseiidae) on tomato. Syst Appl Acarol 26:1264–1279

[CR52] Schmidt-Jeffris RA, Beers EH, Sater C (2021) Meta-analysis and review of pesticide non-target effects on phytoseiids, key biological control agents. Pest Manag Sci 77:4848–4862. 10.1002/ps.6531 34169634 10.1002/ps.6531

[CR47] Sáenz-de-Cabezón Irigaray FJ, Zalom FG, Thompson PB (2007) Residual toxicity of acaricides to Galendromus Occidentalis and *Phytoseiulus Persimilis* reproductive potential. Biol Control 40:153–159. 10.1016/j.biocontrol.2006.10.012 10.1016/j.biocontrol.2006.10.012

[CR54] Shahbaz M, Khoobdel M, Khanjani MJ, Hosseininia A, Khederi SJ (2019) Sublethal effects of acetamiprid on biological aspects and life table of *Amblyseius Swirskii* (Acari: Phytoseiidae) fed on *Aleuroclava Jasmini* (Hemiptera: Aleyrodidae). Syst Appl Acarol 24:814–824

[CR53] Shah RA, Appleby M (2019) Testing the contact and residual toxicity of selected low-risk pesticides to *Tetranychus Urticae* Koch and its predators. SJA 35:1113–1121. 10.17582/journal.sja/2019/35.4.1113.1121 10.17582/journal.sja/2019/35.4.1113.1121

[CR55] Silva DL, Lima JED, Souza PVL, Melo JWS, de Morais Oliveira JE, Lima DB (2023) Selectivity of acaricides to *Neoseiulus barkeri* (Hughes) (Acari: Phytoseiidae). J Appl Entomol 147:1014–1023. 10.1111/jen.13197 10.1111/jen.13197

[CR56] Silva FR, Moraes GJ, Gondim MG Jr., Knapp M, Rouam SL, Paes JL, Oliveira GM (2010) Efficiency of *Phytoseiulus longipes* Evans as a control agent of *Tetranychus evansi* Baker & Pritchard (Acari: Phytoseiidae: Tetranychidae) on screenhouse tomatoes. Neotrop Entomol 39: 991-5. 10.1590/s1519-566x2010000600022 10.1590/s1519-566x2010000600022 21271069

[CR57] Spollen KM, Isman MB (1996) Acute and Sublethal effects of a neem insecticide on the Commercial Biological Control agents *Phytoseiulus persimilis* and *Amblyseius Cucumeris* (Acari: Phytoseiidae) and *Aphidoletes aphidimyza* (Diptera: Cecidomyiidae). J Econ Entomol 89:1379–1386. 10.1093/jee/89.6.1379 10.1093/jee/89.6.1379

[CR58] Stark JD, Banks JE (2003) Population-level effects of pesticides and other toxicants on arthropods. Annu Rev Entomol 48:505–519. 10.1146/annurev.ento.48.091801.112621 12221038 10.1146/annurev.ento.48.091801.112621

[CR59] Sterk G, Hassan SA, Baillod M, Bakker F, Bigler F, Blümel S, Bogenschütz H, Boller E, Bromand B, Brun J, Calis JNM, Coremans-Pelseneer J, Duso C, Garrido A, Grove A, Heimbach U, Hokkanen H, Jacas J, Lewis G, Moreth L, Polgar L, Rovesti L, Samsoe-Peterson L, Sauphanor B, Schaub L, Stäubli A, Tuset JJ, Vainio A, Van de Veire M, Viggiani G, Viñuela E, Vogt H (1999) Results of the seventh joint pesticide testing programme carried out by the IOBC/WPRS-Working Group ‘Pesticides and beneficial organisms’. Biocontrol 44:99–117. 10.1023/A:1009959009802 10.1023/A:1009959009802

[CR60] Uddin N, Alam Z, Miah RU, Mian IH, Mustarin K-E (2015) Toxicity of pesticides to *Tetranychus Urticae* Koch (Acari:Tetranychidae) and their side effects on *Neoseiulus californicus* (Acari:Phytoseiidae). Int J Acarol 41:688–693. 10.1080/01647954.2015.1094512 10.1080/01647954.2015.1094512

[CR61] Ullah MS, Lim UT (2017) Synergism of *Beauveria bassiana* and *phytoseiulus persimilis* in control of *Tetranychus urticae* on bean plants. Syst Appl Acarol 22:1924–1935 12

[CR62] Van de Veire M, Sterk G, van der Staaij M, Ramakers PMJ, Tirry L (2002) Sequential testing scheme for the assessment of the side-effects of plant protection products on the predatory bug *Orius Laevigatus*. Biocontrol 47:101–113. 10.1023/A:1014473023912 10.1023/A:1014473023912

[CR63] Viggiani G, Bernardo U (2001) Side effects of some pesticides on predatory mites (Phytoseiidae) in citrus orchards. In

[CR64] Villanueva RT, Walgenbach JF (2005) Development, oviposition, and mortality of *Neoseiulus fallacis* (Acari: Phytoseiidae) in response to reduced-risk insecticides. J Econ Entomol 98:2114–2120. 10.1093/jee/98.6.2114 16539140 10.1093/jee/98.6.2114

[CR65] Wanumen AC, Carvalho GA, Medina P, Viñuela E, Adán Á (2016) Residual acute toxicity of some modern insecticides toward two mirid predators of tomato pests. J Econ Entomol 109:1079–1085. 10.1093/jee/tow059 27034114 10.1093/jee/tow059

[CR66] Wei M, Chi H, Guo Y, Li X, Zhao L, Ma R (2020) Demography of *Cacopsylla chinensis* (Hemiptera: Psyllidae) reared on four cultivars of *Pyrus bretschneideri* (Rosales: Rosaceae) and *P. communis* pears with estimations of confidence intervals of specific life table statistics. J Econ Entomol 113:2343–2353. 10.1093/jee/toaa149 32785577 10.1093/jee/toaa149

[CR67] Wekesa VW, Moraes GJ, Knapp M, Delalibera I (2007) Interactions of two natural enemies of *Tetranychus Evansi*, the fungal pathogen *neozygites floridana* (zygomycetes: Entomophthorales) and the predatory mite, *Phytoseiulus longipes* (Acari: Phytoseiidae). Biol Control 41:408–414. 10.1016/j.biocontrol.2007.03.003 10.1016/j.biocontrol.2007.03.003

[CR68] Yaninek JS, Megevand B, Ojo B, Cudjoe AR, Abole E, Onzo A, Zannou I (1998) Establishment and spread of *Typhlodromalus manihoti* (Acari: Phytoseiidae), an Introduced Phytoseiid Predator of *Mononychellus tanajoa* (Acari: Tetranychidae) in Africa. Environ Entomol 27:1496–1505. 10.1093/ee/27.6.1496 10.1093/ee/27.6.1496

[CR69] Zacarias MS, Da Silveira EC, De Oliveira Bernardi LF (2019) Predator mites.(pp89-96). Springer International Publishing

[CR70] Zanardi OZ, Pavan Bordini G, Aparecida Franco A, Jacob CRO (2017) Sublethal effects of pyrethroid and neonicotinoid insecticides on *Iphiseiodes Zuluagai* Denmark and Muma (Mesostigmata: Phytoseiidae). Ecotoxicology 26:1188–1198 28819698 10.1007/s10646-017-1844-x

[CR71] Zemek R, Prenerová E, Volter L, Awad M, Weyda F, Hussein H, Skoková OH Půža V (2017) Non-target impacts of *Isaria fumosorosea* (Hypocreales: Cordycipitaceae) on natural enemies of arthropod pests. In: Proceedings of the 5th International Symposium on Biological Control of Arthropods, Langkawi, Malaysia, September 11–15, 2017. CABI Wallingford UK, p 294–296

